# Supplementary figures and images for: Mck1 defines a key S-phase checkpoint effector in response to various degrees of replication threats
Source: PLoS Genet. 2019 Aug 5;15(8):e1008136. doi: 10.1371/journal.pgen.1008136 (PMC6695201; doi:10.1371/journal.pgen.1008136)

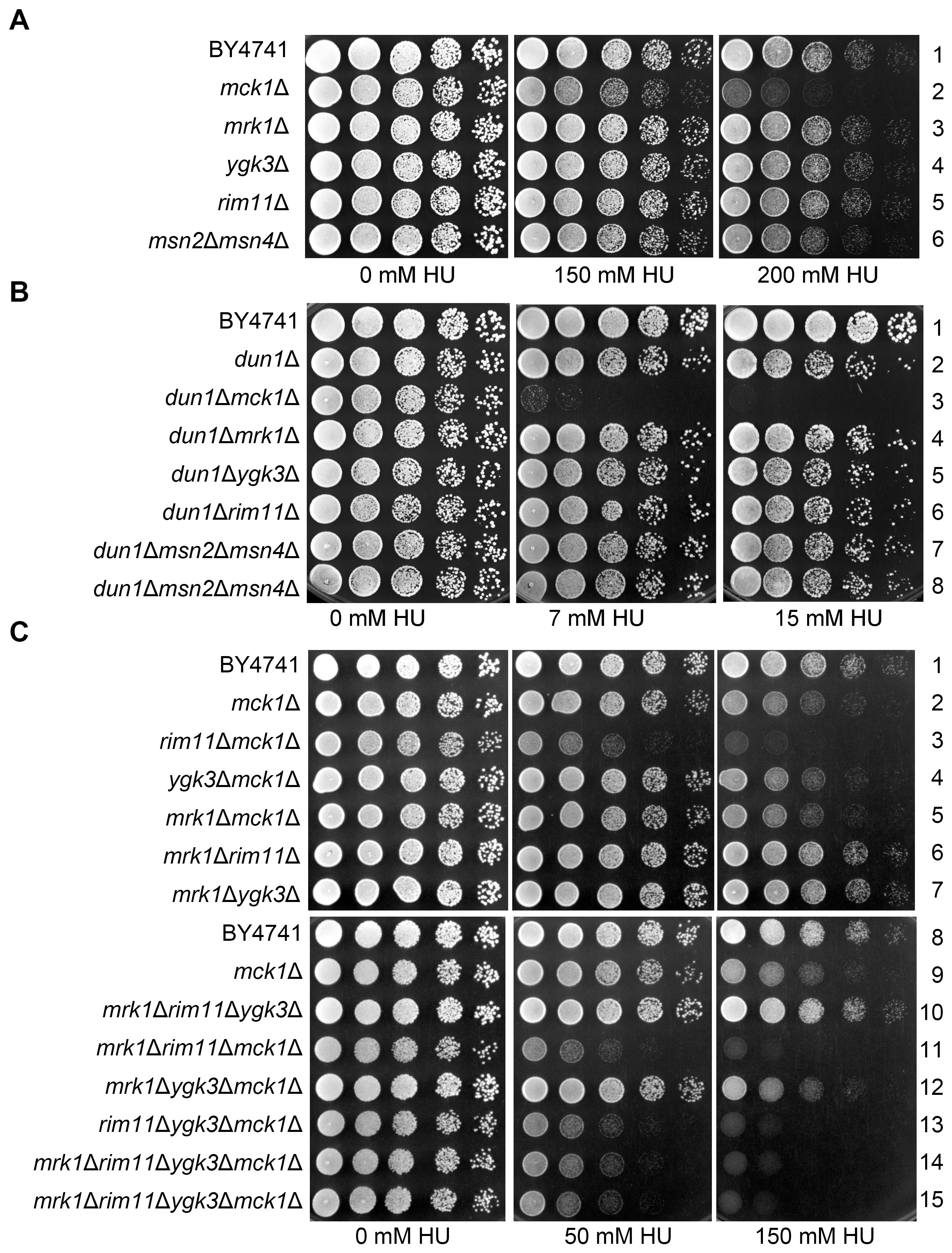

Supplement: S1 Fig — A) Among GSK-3 family kinases, only MCK1 deletion exhibits HU sensitivity. WT, mck1Δ, mrk1Δ, ygk3Δ, rim11Δ, msn2Δmsn4Δ (S1 Table) were tested for the HU sensitivity by serial dilution analysis as described in Fig 1A. B) Among GSK-3 family kinases, only MCK1 deletion shows synthetic HU sensitivity with dun1Δ. WT, dun1Δ, dun1Δmck1Δ, dun1Δmrk1Δ, dun1Δygk3Δ, dun1Δrim11Δ, dun1Δmsn2Δmsn4Δ (S1 Table) were tested for the HU sensitivity by serial dilution analysis as described in Fig 1A. C) RIM11 deletion, but neither MRK1 nor YGK3 deletion, shows synthetic HU sensitivity with mck1Δ. Yeast strains with the indicated genotype (S1 Table) were tested for the HU sensitivity by serial dilution analysis as described in Fig 1A. (TIF) [file pgen.1008136.s001.tif]

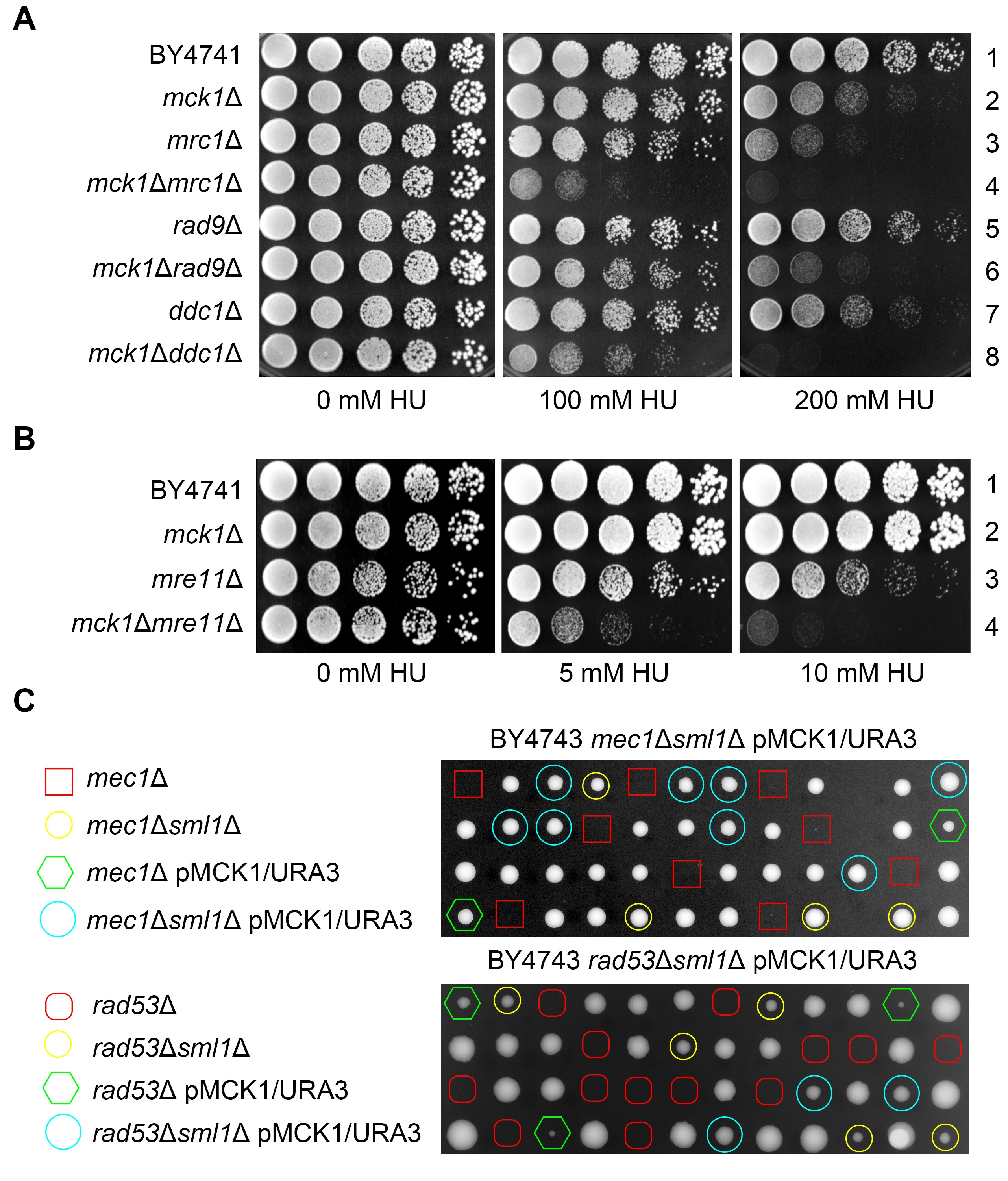

Supplement: S2 Fig — (A, B) MCK1 deletion shows synthetic HU sensitivity with mrc1Δ, rad9Δ, ddc1Δ or mre11Δ. Yeast strains with the indicated genotype (S1 Table) were tested for the HU sensitivity by serial dilution analysis as described in Fig 1A. (C) MCK1 overexpression is able to bypass the essentiality of MEC1 and RAD53. Representative tetrad dissection analyzed using the diploid cells with the indicated genotype. (TIF) [file pgen.1008136.s002.tif]

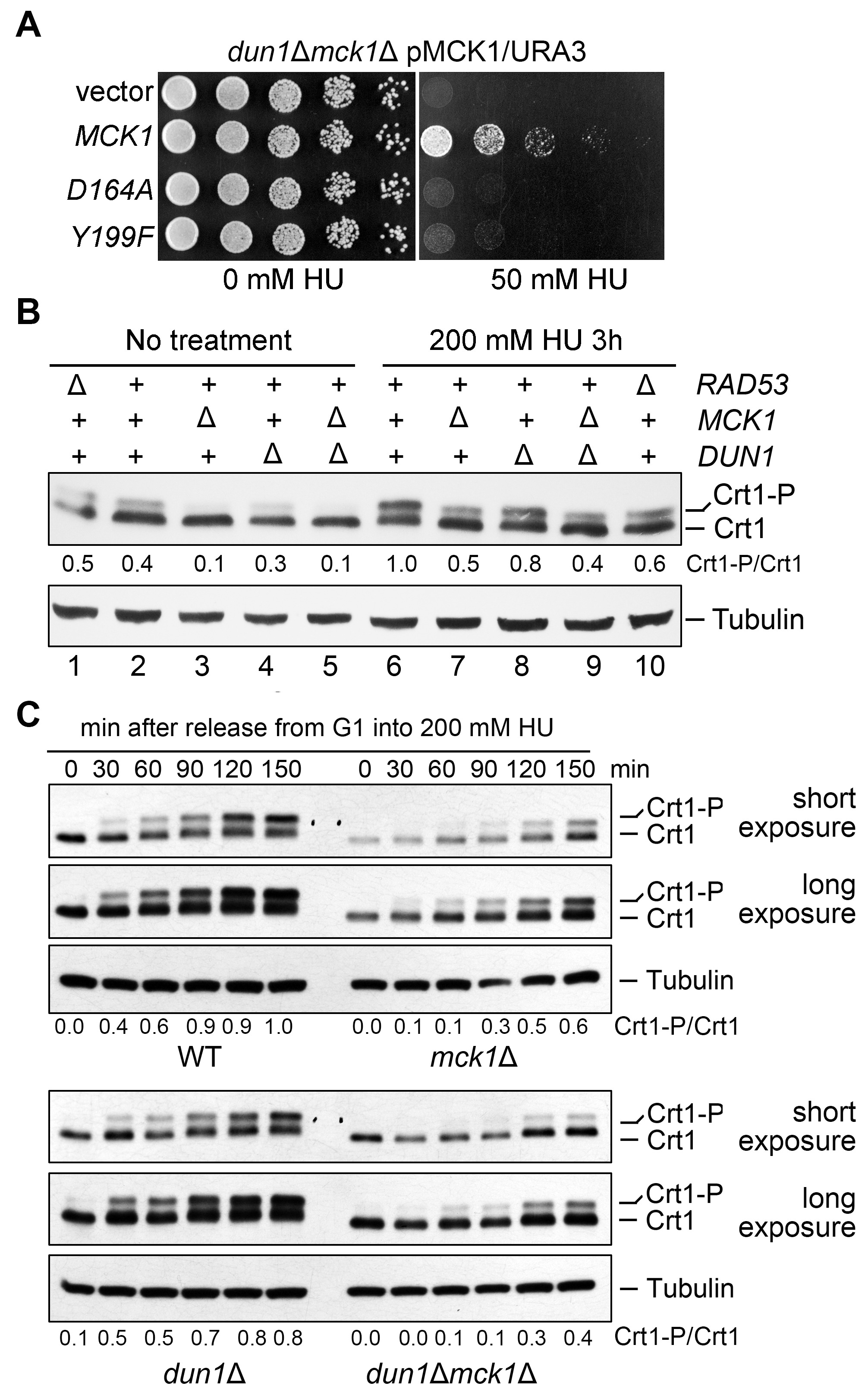

Supplement: S3 Fig — (A) Mck1 acts as a kinase in response to HU. The dun1Δmck1Δ strain was transformed with pRS316 empty vector, WT MCK1 or mck1 alleles (the catalytic mutant allele, D164A; the activation-loop mutant allele, Y199F). Strains were spotted onto SC-Ura media with or without 50 mM HU and grown at 30°C for 48 h. (B) Mck1 affects Crt1 phosphorylation. Cells were grown to the stationary phase. Lysates were prepared and resolved by a 7% polyacrylamide (acrylamide: N’N’-bis-methylene-acrylamide = 149:1) gel containing SDS. The phosphorylation of Crt1-13Myc was detected by immunoblots using an anti-Myc antibody. Tubulin was applied as a loading control. (C) Biological repeats of Fig 3C. (TIF) [file pgen.1008136.s003.tif]

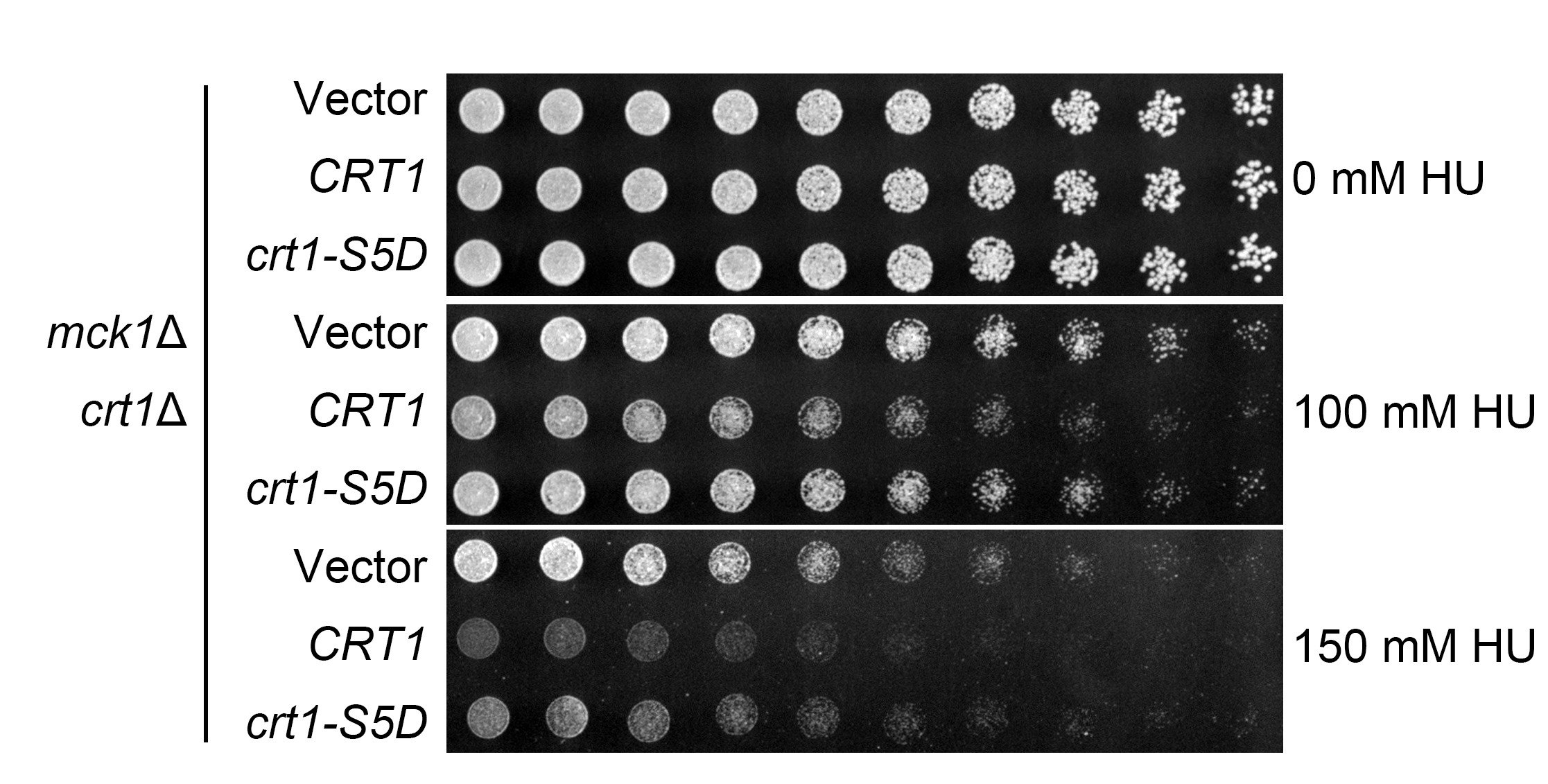

Supplement: S4 Fig — crt1-5D rescues the HU sensitivity of mck1Δ. The mck1Δcrt1Δ strain was transformed with pRS313 empty vector, WT CRT or crt1-5D mutant. Two-fold serial dilution of the cells was spotted onto SC-His media with or without HU. (TIF) [file pgen.1008136.s004.tif]

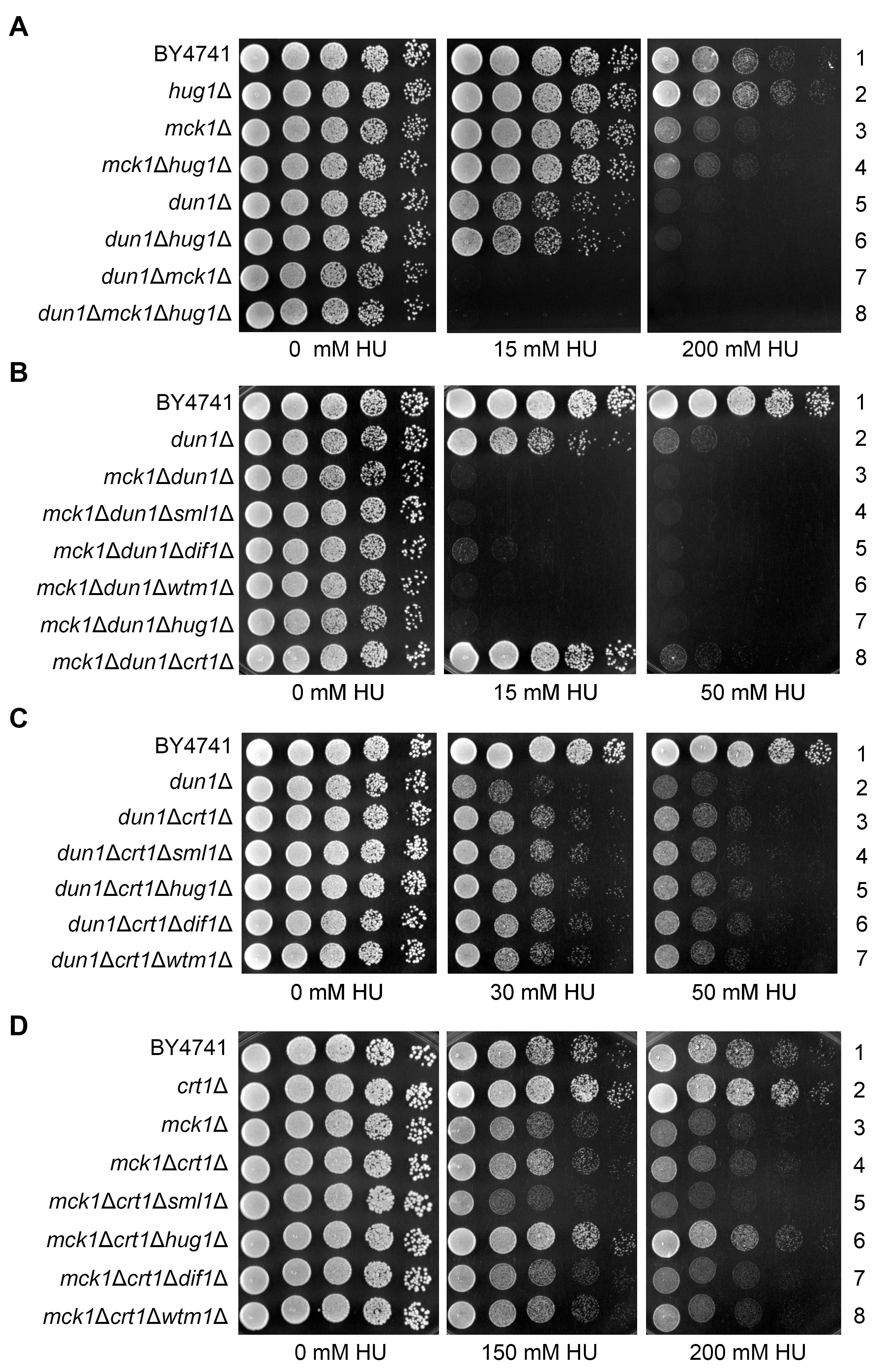

Supplement: S5 Fig — A) HUG1 deletion shows no synthetic sensitivity with mck1Δ, dun1Δ or dun1Δmck1Δ. WT, hug1Δ, mck1Δ, mck1Δhug1Δ, dun1Δ, dun1Δhug1Δ, dun1Δmck1Δ, dun1Δmck1Δhug1Δ (S1 Table) were tested for the HU sensitivity by serial dilution analysis as described in Fig 1A. B) The deletion of CRT1, but not of SML1, HUG1, DIF1 or WTM1, shows suppression in mck1Δdun1Δ. WT, dun1Δ, dun1Δmck1Δ, dun1Δmck1Δsml1Δ, dun1Δmck1Δdif1Δ, dun1Δmck1Δwtm1Δ, dun1Δmck1Δhug1Δ, dun1Δmck1Δcrt1Δ (S1 Table) were tested for the HU sensitivity by serial dilution analysis as described in Fig 1A. C) Removal of SML1, HUG1, DIF1 or WTM1 has no detectable effects with crt1Δdun1Δ. Yeast strains with the indicated genotype (S1 Table) were tested for the HU sensitivity by serial dilution analysis as described in Fig 1A. D) Removal of HUG1, but not SML1, DIF1 or WTM1, had a rescue on crt1Δmck1Δ. Yeast strains with the indicated genotype (S1 Table) were tested for the HU sensitivity by serial dilution analysis as described in Fig 1A. (TIF) [file pgen.1008136.s005.tif]
