# Supplementary material for: Mck1 defines a key S-phase checkpoint effector in response to various degrees of replication threats
Source: PLoS Genet. 2019 Aug 5;15(8):e1008136. doi: 10.1371/journal.pgen.1008136 (PMC6695201; doi:10.1371/journal.pgen.1008136)
Supplement: S1 Table — (DOCX) [file pgen.1008136.s006.docx]

**S1 Table. Strains used in this study.**

| Lab name | Genotype | Resource and Used for |
| --- | --- | --- |
| BY4741 | *MATa his3*Δ*1 leu2*Δ*0 met15*Δ*0 ura3*Δ*0 lys2*Δ*0* | In stock |
| AH109 | *MATa trp1-901 leu2-3,112 ura3-53 his3-200 gal4*Δ *gal80*Δ *LYS2::GAL1 UAS -GAL1 TATA -HIS3 GAL2 UAS -GAL1 TATA -ADE2 URA3::MEL1 UAS -MEL1 TATA -lacZ MEL1* | Fig 1D |
| LXL160708002 | *BY4741 mck1*Δ*::NatMX* | Fig1B,2A,2D,2E,4F,4H,5A,6B,6C,7A,S1A,S1C,S2A,S2B,S5A,S5D |
| LXL160708003 | *BY4741dun1*Δ*::NatMX* | Fig1B,2D,2E,4F,4H,6B,6C,7A,S1B,S3A,S5A,S5B,S5C |
| LXL160708004 | *BY4741dun1*Δ*::NatMX mck1*Δ*::HygR* | Fig1B,2D,2E,4F,4H,6B,6C,7A,S1B,S5A,S5B |
| LXL160708005 | *BY4741mrk1*Δ*::NatMX* | FigS1A |
| LXL160708006 | *BY4741dun1*Δ*::NatMX mrk1*Δ*::HygR* | FigS1B |
| LXL160708007 | *BY4741 gsk3*Δ*::LEU2* | FigS1A |
| LXL160708008 | *BY4741 dun1*Δ*::NatMX gsk3*Δ*::LEU2* | FigS1B |
| LXL160708009 | *BY4741 rim11*Δ*::LEU2* | FigS1A |
| LXL160708010 | *BY4741 dun1*Δ*::NatMX rim11*Δ*::LEU2* | FigS1B |
| LXL160708013 | *BY4741 msn2*Δ*::* *LEU2 msn4*Δ*::URA3* | FigS1A |
| LXL160708016 | *BY4741 dun1*Δ*::NatMX msn2*Δ*::LEU2 msn4*Δ*::URA3* | FigS1B |
| LXL160930060 | *BY4741 crt1*Δ*:: LEU2* | Fig2D,5A,5B,6B |
| LXL160930061 | *BY4741 mck1*Δ*::NatMX crt1*Δ*::LEU2* | Fig2D,5B,6B,S4 |
| LXL160930062 | *BY4741 dun1*Δ*::NatMX crt1*Δ*::LEU2* | Fig2D,2E,5B,6B |
| LXL160930063 | *BY4741 dun1*Δ*::NatMX mck1*Δ*::HygR crt1*Δ*::LEU2* | Fig2D,4B,5B,5D,6B,7A |
| LXL150917001 | *BY4741 CRT1-13Myc::HIS3* | Fig3A,3B,3C,3D,S3B,S3C |
| LXL150917003 | *BY4741 mck1*Δ*::KanMX6 CRT1-13Myc::HIS3* | Fig3A,3B,3C,3D,S3B,S3C |
| LXL150917005 | *BY4741 dun1*Δ*::NatMx CRT1-13Myc::HIS3* | Fig3A,3B,3C,3D,S3B,S3C |
| LXL150917006 | *BY4741 dun1*Δ*::NatMx mck1*Δ*::KanMX6 CRT1-13Myc::HIS3* | Fig3A,3B,3C,3D,S3B,S3C |
| LXL160930058 | *BY4741 rad53*Δ*::KanMX6 sml1*Δ*::LEU2 CRT1-13Myc::HIS3* | FigS3B |
| LXL160930064 | *BY4741 RNR3-13Myc::HIS3* | Fig4G |
| LXL160930065 | *BY4741 mck1*Δ*::NatMx RNR3-13Myc::HIS3* | Fig4G |
| LXL160930067 | *BY4741 dun1*Δ*::NatMx RNR3-13Myc::HIS3* | Fig4G |
| LXL160930069 | *BY4741 dun1*Δ*::NatMx mck1*Δ*::HphMx RNR3-13Myc::HIS3* | Fig4G |
| LXL160930099 | *BY4741 HUG1-13Myc::HIS3* | Fig6A |
| LXL160930101 | *BY4741 mck1*Δ*::NatMx HUG1-13Myc::HIS3* | Fig6A |
| LXL160930103 | *BY4741 dun1*Δ*::NatMx HUG1-13Myc::HIS3* | Fig6A |
| LXL160930105 | *BY4741 dun1*Δ*::NatMx mck1*Δ*::HphMx HUG1-13Myc::HIS3* | Fig6A |
| LXL160930106 | *BY4741 crt1*Δ*::LEU2 HUG1-13Myc::HIS3* | Fig6A |
| LXL160930108 | *BY4741 mck1*Δ*::NatMx crt1*Δ*::LEU2 HUG1-13Myc::HIS3* | Fig6A |
| LXL160930110 | *BY4741 dun1*Δ*::NatMx crt1*Δ*::LEU2 HUG1-13Myc::HIS3* | Fig6A |
| LXL160930112 | *BY4741 dun1*Δ*::NatMx mck1*Δ*::HphMx crt1*Δ*::LEU2 HUG1-13Myc::HIS3* | Fig6A |
| LXL160930006 | *BY4741 mec1*Δ*:: KanMX6 sml1*Δ*::LEU2* | Fig1A,6D |
| LXL160930011 | *BY4741 mec1*Δ*:: KanMX6 sml1*Δ*::LEU2 crt1*Δ*:: HygR* | Fig6D |
| LXL160930012 | *BY4741 mec1*Δ*:: KanMX6 crt1*Δ*:: HygR* | Fig6D |
| LXL160930120 | *BY4741 mec1*Δ*:: KanMX6 sml1*Δ*::LEU2 hug1*Δ*::NatMx* | Fig6D |
| LXL160930121 | *BY4741 mec1*Δ*:: KanMX6 sml1*Δ*::LEU2 hug1*Δ*::NatMx crt1*Δ*:: HygR* | Fig6D |
| LXL180120047 | *BY4741 dun1*Δ*::NatMx mck1*Δ*::HygR crt1*Δ*::LEU2* TAG-*HUG1* | Fig5D |
| LXL180120038 | *BY4741* YFP-Sml1*:: KanMX6* | Fig2C |
| LXL180120039 | *BY4741 mck1*Δ*::NatMx* YFP-Sml1*:: KanMX6* | Fig2C |
| LXL190610003 | *BY4741 mck1*Δ*:: NatMx rim11*Δ*:: LEU2* | FigS1C |
| LXL190610004 | *BY4741 mck1*Δ*:: NatMx ygk3*Δ*:: LEU2* | FigS1C |
| LXL190610006 | *BY4741 mck1*Δ*:: NatMx mrk1*Δ*::HIS3* | FigS1C |
| LXL190610007 | *BY4741 mrk1*Δ*::HIS3 rim11*Δ*::URA3* | FigS1C |
| LXL190610008 | *BY4741 mrk1*Δ*::HIS3 ygk3*Δ*::URA3* | FigS1C |
| LXL190610009 | *BY4741 mck1*Δ*:: NatMx mrk1*Δ*::HIS3 rim11*Δ*::URA3* | FigS1C |
| LXL190610010 | *BY4741 mck1*Δ*:: NatMx mrk1*Δ*::HIS3 ygk3*Δ*::URA3* | FigS1C |
| LXL190610012 | *BY4741 mck1*Δ*:: NatMx ygk3*Δ*::LEU2 rim11*Δ*::HIS3* | FigS1C |
| LXL190610021 | *BY4741 mrk1*Δ*:: HIS3 ygk3*Δ*::URA3 rim11*Δ*::LEU* | FigS1C |
| LXL190610023 | *BY4741 mck1*Δ*:: NatMx mrk1*Δ*:: HIS3 ygk3*Δ*::URA3 rim11*Δ*::LEU* | FigS1C |
| ZJXY010 | *BY4741 dun1*Δ*::NatMx mck1*Δ*::HygR crt1*Δ*::LEU2 hug1*Δ*::URA3* | Fig5B,5D, |
| ZJXY013 | *BY4741 dun1*Δ*::NatMx crt1*Δ*::LEU2 sml1*Δ*::URA3* | FigS5C |
| ZJXY014 | *BY4741 dun1*Δ*::NatMx crt1*Δ*::LEU2 hug1*Δ*::URA3* | Fig5B,S5C |
| ZJXY015 | *BY4741 dun1*Δ*::NatMx crt1*Δ*::LEU2 dif1*Δ*::URA3* | FigS5C |
| ZJXY016 | *BY4741 dun1*Δ*::NatMx crt1*Δ*::LEU2 wtm1*Δ*::URA3* | FigS5C |
| ZJXY018 | *BY4741 hug1*Δ*::URA3* | Fig5C,S5A |
| ZJXY022 | *BY4741 dun1*Δ*::NatMx hug1*Δ*::URA3* | FigS5A |
| ZJXY026 | *BY4741 mck1*Δ*::NatMx hug1*Δ*::URA3* | Fig5A,S5A |
| ZJXY033 | *BY4741 dun1*Δ*::NatMx mck1*Δ*::HygR sml1*Δ*::URA3* | FigS5B |
| ZJXY034 | *BY4741 dun1*Δ*::NatMx mck1*Δ*::HygR hug1*Δ*::URA3* | FigS5B |
| ZJXY035 | *BY4741 dun1*Δ*::NatMx mck1*Δ*::HygR dif1*Δ*::URA3* | FigS5B |
| ZJXY036 | *BY4741 dun1*Δ*::NatMx mck1*Δ*::HygR wtm1*Δ*::URA3* | FigS5B |
| ZJXY037 | *BY4741 mck1*Δ*::NatMx crt1*Δ*::LEU2 sml1*Δ*::URA3* | Fig5A |
| ZJXY038 | *BY4741 mck1*Δ*::NatMx crt1*Δ*::LEU2 hug1*Δ*::URA3* | Fig5A |
| ZJXY039 | *BY4741 mck1*Δ*::NatMx crt1*Δ*::LEU2 dif1*Δ*::URA3* | Fig5A |
| ZJXY040 | *BY4741 mck1*Δ*::NatMx crt1*Δ*::LEU2 wtm1*Δ*::URA3* | Fig5A |
