# Supplementary material for: Mck1 defines a key S-phase checkpoint effector in response to various degrees of replication threats
Source: PLoS Genet. 2019 Aug 5;15(8):e1008136. doi: 10.1371/journal.pgen.1008136 (PMC6695201; doi:10.1371/journal.pgen.1008136)
Supplement: S2 Table — (DOCX) [file pgen.1008136.s007.docx]

**S2 Table. Plasmids used in this study**

| Plasmid | Base plasmid/Genotype | Used for |
| --- | --- | --- |
| pRS313-p-CRT1-13Myc | *amp^r^/HIS3 CRT1-13Myc* | Fig4B,4C,4D,4E,S4 |
| pRS313-p-*crt1-S58*,*62D-13Myc* | *amp^r^/HIS3 crt1-S58*,*62D-13Myc* | Fig4B |
| pRS313-p-*crt1-S167*,*171*,*173*,*174D-13Myc* | *amp^r^/HIS3 crt1-S167*,*171*,*173*,*174D-13Myc* | Fig4B |
| pRS313-p-*crt1-T197*,*199D-13Myc* | *amp^r^/HIS3 crt1-T197*,*199D-13Myc* | Fig4B |
| pRS313-p-*crt1-S222*,*T226D-13Myc* | *amp^r^/HIS3 crt1-S222*,*T226D-13Myc* | Fig4B |
| pRS313-p-*crt1-S295*,*299D-13Myc* | *amp^r^/HIS3 crt1-S295*,*299D-13Myc* | Fig4B,4C,4D,4E,S4 |
| pRS313-p-*crt1-S295*,*299A-13Myc* | *amp^r^/HIS3 crt1-S295*,*299A-13Myc* | Fig4C,4D,4E |
| pRS313-p-*crt1-S295D-13Myc* | *amp^r^/HIS3 crt1-S295D-13Myc* | Fig4B |
| pRS313-p-*crt1-S299D-13Myc* | *amp^r^/HIS3 crt1-S299D-13Myc* | Fig4B |
| pRS313-p-*crt1-S323D-13Myc* | *amp^r^/HIS3 crt1-S323D-13Myc* | Fig4B |
| pRS313-p-*crt1-S388*,*389*,*391*,*393*,*394D-13Myc* | *amp^r^/HIS3 crt1-S388*,*389*,*391*,*393*,*394D-13Myc* | Fig4B |
| pRS313-p-*crt1-S412*,*S414*,*T416*,*S418D-13Myc* | *amp^r^/HIS3 crt1-S412*,*S414*,*T416*,*S418D-13Myc* | Fig4B |
| pRS313-p-*crt1-T488*,*S492D-13Myc* | *amp^r^/HIS3 crt1- T488*,*S492D-13Myc* | Fig4B |
| pRS313-p-*crt1-S556*,*558*,*560*,*562D-13Myc* | *amp^r^/HIS3 crt1-S556*,*558*,*560*,*562D-13Myc* | Fig4B |
| pGADT7-RAD53 | *amp^r^/LEU2 GAL4-AD-RAD53* | Fig1D |
| pGADT7-*RAD53-FHA1* | *amp^r^/LEU2 GAL4-AD-rad53(1-300)* | Fig1D |
| pGADT7-*RAD53-FHA2* | *amp^r^/LEU2 GAL4-AD-rad53(483-C)* | Fig1D |
| pGBKT7-MCK1 | *kan^r^/TRP1 GAL4-BD-MCK1* | Fig1D |
| pGBKT7-*mck1-T218*,*219A* | *kan^r^/TRP1 GAL4-BD-mck1-T218*,*219A* | Fig1D |
| pGBKT7-*mck1-T121*,*131*,*145*,*176*,*218*,*219*,*342A* | *kan^r^/TRP1 GAL4-BD-mck1-T121*,*131*,*145*,*176*,*218*,*219*,*342A* | Fig1D |
| pRS313-pADH1-MCK1-5FLAG | *Amp^r^/HIS3 pADH1-MCK1-5FLAG* | Fig1E,4A |
| pRS313-pADH1-*MCK1-KD*-5FLAG | *Amp^r^/HIS3 pADH1-MCK1-KD(D164A)-5FLAG* | Fig1E,4A |
| pET-15b-RAD53 | *amp^r^ 6His-RAD53* | Fig1E |
| pET-15b-*rad53-KD(K227A)* | *amp^r^ 6His-rad53-KD(K227A)* | Fig1E |
| pRS316-p-MCK1 | *amp^r^/HIS3 MCK1* | FigS3A |
| pRS316-p-*mck1-D164A* | *amp^r^/HIS3 mck1-D164A* | FigS3A |
| pRS316-p-*mck1-Y199F* | *amp^r^/HIS3 mck1-Y199F* | FigS3A |
| pET-28a-CRT1(200-453) | *kan^r^ 6His-CRT1(201-453)* | Fig4A |
| pET-28a-*CRT1(200-453)-S299D* | *kan^r^ 6His-CRT1(201-453)-S299D* | Fig4A |
| pET-28a-DUN1 | *kan^r^ 6His-DUN1* | Fig4A |
